# Pancreatoduodenectomy with or without prophylactic falciform ligament wrap around the gastroduodenal artery stump for prevention of pancreatectomy hemorrhage

**DOI:** 10.1186/s13063-018-2580-0

**Published:** 2018-04-12

**Authors:** Benjamin Müssle, Leonie Zühlke, Ann Wierick, Dorothée Sturm, Xina Grählert, Marius Distler, Nuh N. Rahbari, Jürgen Weitz, Thilo Welsch

**Affiliations:** 1 Department of Visceral, Thoracic and Vascular Surgery, University Hospital Carl Gustav Carus, Technische Universität, Dresden, Germany; 2 Coordination Centre for Clinical Trials, Faculty of Medicine Carl Gustav Carus, Technische Universität, Dresden, Germany

**Keywords:** Falciform ligament, Pancreatoduodenectomy, Pancreatic fistula, Hemorrhage, Gastroduodenal artery

## Abstract

**Background:**

The purpose of this study is to evaluate whether wrapping of the pedicled falciform ligamentum flap around the gastroduodenal artery (GDA) stump/hepatic artery can significantly decrease the incidence of erosion hemorrhage after pancreatoduodenectomy (PD).

**Methods/design:**

This is a randomized controlled multicenter trial involving 400 patients undergoing PD. Patients will be randomized into two groups. The intervention group consists of 200 patients with a prophylactic wrapping of the GDA stump using the pedicled falciform ligament. The control group consists of 200 patients without the wrap. The primary endpoint is the rate of postoperative erosion hemorrhage of the GDA stump or hepatic artery within 3 months. The secondary endpoints are postpancreatectomy hemorrhage stratified according to the texture of the pancreas, postoperative pancreatic fistula (POPF), postoperative rate of therapeutic interventions, morbidity, and mortality.

**Discussion:**

Only few retrospective studies investigated the effectiveness of a falciform ligament wrap around the GDA for prevention of erosion hemorrhage. Erosion hemorrhage occurs in up to 6–9% of cases after PD and is most frequently evoked by a POPF. Erosion hemorrhage is associated with a remarkable mortality of over 30%. The rate of hemorrhage after performing the wrap is reported to be low. However, there exist no prospectively controlled data to support its general use. Therefore, the presented randomized controlled trial will provide clinically relevant evidence of the effectiveness of the wrap with statistical significance.

**Trial registration:**

clinicaltrials.gov, NCT02588066; Registered on 27 October 2015.

**Electronic supplementary material:**

The online version of this article (10.1186/s13063-018-2580-0) contains supplementary material, which is available to authorized users.

## Background

Pancreatoduodenectomy (PD) is the standard surgical procedure for malignant, benign, and borderline tumors (e.g., intraductal papillary mucinous neoplasms) of the pancreatic head and neck. Moreover, it is also indicated for some patients with chronic pancreatitis. Postoperative morbidity has been reduced by centralization at high-volume centers, advances in surgical techniques, and perioperative management but still ranges between 40 and 60% [1]. The most frequent surgical postoperative complications are development of a pancreatic fistula (POPF), delayed gastric emptying (DGE), and postpancreatectomy hemorrhage (PPH) [2]. The development of a POPF is one of the most complex complications after PD [3]. The incidence of POPF still ranges between 20 and 30% after pancreatic resections [4, 5]. The most serious sequela of an established POPF is the erosion of the gastroduodenal artery (GDA) stump by pancreatic juice or local inflammation, leading to delayed and potentially lethal PPH. Erosion hemorrhage occurs with the usual delay of a few days up to several weeks [6–8]. Late PPH is predominantly caused by erosion or pseudoaneurysm formation of the GDA or hepatic artery (HA) (>24 h after the index operation). According to the literature, the incidence ranges between 3 and 6% or even higher in single-center studies and is associated with a significantly increased mortality of 16–20% [7–9].

In recent years, technical efforts have been undertaken to lower the incidence of erosion hemorrhage or POPF, i.e., covering of either the skeletonized and divided arteries or the pancreatojejunostomy with the round or falciform ligament or with an omental flap [10]. A promising surgical technique for covering the GDA stump is to wrap the pedicled falciform ligament around the skeletonized HA and the GDA stump [11]. This procedure is frequently practiced in Asia, although its routine use lacks solid evidence and is rarely used in Europe or the United States [10]. There are no reports on randomized controlled trials investigating the prophylactic use of the pedicled falciform ligament for prevention of erosion hemorrhage [12]. For this reason, the presented randomized controlled multicenter trial was initiated to assess whether the use of the pedicled falciform ligament wrap can significantly decrease the incidence of postpancreatectomy erosion hemorrhage.

## Methods/design

### Administrative information

The trial was registered at ClinicalTrials.gov (NCT02588066) on October 27, 2015. The trial is initiated by the Department of Visceral, Thoracic and Vascular Surgery, University Hospital Carl Gustav Carus, Technische Universität Dresden, Germany. Funding for this trial covers meetings and central organizational costs only; there is no third-party funding support for this trial. The contact information of the head of the clinical trial is as follows: Fetscherstr. 74, 01307 Dresden, Germany (phone: +49 351 458 2742, fax: +49 351 458 7240, email: klinikportal-vtg@uniklinikum-dresden.de). The head of the trial is the senior author of this study protocol. All participating centers have to sign a collaboration contract, which regulates responsibilities, ownership, and publication issues with the head of the clinical trial.

### Trial design and study setting

The title of this clinical trial is “Pancreatoduodenectomy with or without prophylactic falciform ligament wrap around the gastroduodenal artery stump for prevention of pancreatectomy hemorrhage” (Additional file 1). The study is designed as a randomized controlled, national (Germany) multicenter trial with an interventional group (A: PD operation with the creation of a pedicled falciform ligament wrap around the GDA stump) and a control group (B: PD operation without the ligament wrap). Participating centers are at least four German university centers and two academic centers, which are officially certified for pancreatic surgery (pancreatic cancer center). Immediate access to computed tomography (CT) and a 24-h availability of interventional radiology must be guaranteed. A list of participating centers can be obtained from the senior author by request.

### Aim of the study and study endpoints

The aim of the study is to evaluate whether a pedicled falciform ligament wrap can decrease erosion hemorrhage of the stump of the GDA in the presence of a POPF.

Therefore, the primary endpoint of the study is the rate of postoperative erosion hemorrhage from the GDA stump or the HA within 3 months from the index operation.

The secondary endpoints are:

- Incidence of late PPH (according to the International Study Group of Pancreatic Surgery (ISGPS) definition [13]) in the entire study population and divided into subgroups with soft and hard pancreatic texture (also depending on the underlying histopathology [e.g., cancer, cystic lesion, or chronic pancreatitis])
- Incidence of clinically relevant POPF (according to POPF grades B and C, ISGPS definition [3, 14])
- Incidence of symptomatic hepatic malperfusion and narrowing/stenosis of the HA (diagnosed by computed tomography or angiography)
- Postoperative rate of therapeutic interventions (computed tomography-guided drainage or angiographic studies)
- Reoperation rate
- Postoperative morbidity and mortality (during hospital stay and at 3 months postoperative)

Erosion hemorrhage from the GDA stump or the HA is defined as postoperative bleeding or pseudoaneurysm formation (proved by CT angiography, conventional angiography, or relaparotomy) from the GDA stump or the HA within 3 months from the index operation. Clinically relevant POPF grade C is defined as POPF, which is causative for a reoperation, organ failure, or death of the patient [14].

### Study population (inclusion and exclusion criteria)

The targeted study population includes all patients scheduled for elective open PD (Whipple or pylorus-preserving) with reconstruction using a pancreatojejunostomy (anastomosis of the pancreas to the jejunum) in cases of tumors or cystic lesions of the pancreatic head, the distal bile duct, and the duodenum or in cases of chronic pancreatitis. Further inclusion criteria are as follows: male and female patients, age equal to or older than 18 years, American Society of Anesthesiologists (ASA) score I–III, and a completed written informed consent form. Exclusion criteria are as follows: potential conditions/circumstances after previous abdominal surgery with resection of the falciform ligament (e.g., status post liver resection), no creation of a pancreatojejunostomy (e.g., pancreatogastrostomy, total pancreatectomy, or non-resectability), and simultaneous arterial resection or reconstruction (e.g., hepatic or splenic, or superior mesenteric artery).

### Surgical technique

The standard technique of PD and surgical instruments may vary in several aspects. The technique and critical steps of the own Dresden center were described elsewhere [15].

The following operation steps are crucial for the trial evaluation and are predefined for patients in both the intervention and control arms:

- The abdominal incision can be either a midline laparotomy or a transverse subcostal incision.
- After opening the abdominal cavity, attention should be paid to preserve the round and falciform ligament over its entire length, before division near the umbilicus and separation from the ventral abdominal wall.
- Preparation of the falciform ligament (see also [12]): The falciform ligament is then further dissected from the ventral abdominal cephalad along the ventral attachment. At the junction to the coronary ligament, the falciform ligament is freed from the ventral hepatic surface until the round ligament is reached. This technique results in a pedicled falciform ligament, with the round ligament being the pedicle.
- When dividing the GDA, the stump of the GDA should be kept as long as possible. Division of the GDA is performed using a suture stitch (Prolene 4-0) and two titanium clips as a standard. Other deviating techniques of dividing the GDA are allowed according to the protocol but should be recorded in the case report form (CRF).
- Reconstruction is achieved by an end-to-side pancreatojejunostomy. The technique of this type of anastomosis is at the discretion of the surgeon.
- A resection of the portal vein can be performed if necessary and is no exclusion criterion.
- Intra-abdominal drains can be placed after completion of the operation.

For patients in the intervention group, the technique for creation of the pedicled falciform ligament wrap as it should be performed in this trial is as follows: After completion of the pancreatic, bile duct, or gastric/duodenal anastomosis, the prepared pedicled falciform ligament is carefully tunneled below the common HA and wrapped around the GDA stump in a tension-free fashion using only one turn. Fixation is then performed with two to three stitches using polydioxanone (PDS) 5-0. The last step is to ensure a proper pulsation of the HA after completion of the wrap. The covering of the divided and skeletonized arteries (GDA stump, hepatic artery) by the pedicled falciform ligament patch is a quick and safe surgical procedure for experienced pancreatic surgeons.

### Randomization

Patients will be screened for eligibility considering the inclusion criteria (see above) on the day of admission (usually the day before the surgery). After obtaining the written informed consent, the randomization will be performed intraoperatively after proving the exclusion criteria. The randomization is designed as block randomization (via envelopes), with fixed block sizes in a 1:1 allocation ratio. The envelopes were prepared by an authorized trial coordinator at the center in Dresden and distributed to the participating sites as needed. It is mandatory to check all eligibility criteria before opening of a randomization envelope by authorized trial personnel only. The investigator has to consecutively assign the envelopes to the patients. He is requested to document the assignment carefully in the patient identification log.

The details of the randomization will be kept safe and confidential. Subjects withdrawn from the trial retain their identification codes (e.g., randomization number). New subjects receive a new identification code.

The randomization sequence was generated using the established R statistics software package (R version 3.1.3, the R Foundation for Statistical Computing). The block size will be kept confidential until completion of recruitment. Eligible patients will be randomized intraoperatively to one of the two groups (control group or intervention group) after the surgeon has confirmed resectability and the availability of the pedicled falciform ligament. In cases when exclusion criteria (e.g., no performance of a pancreatojejunostomy or total pancreatectomy) are met after randomization, the respective patients were withdrawn from the trial.

### Study visits and data collection

The trial includes a total of five study visits during the operation or the postoperative period. The period ranges from the day of the operation until 3 months after the operation (Table 1). All outcome parameters will be recorded by a surgical resident or fellow before and after the operation, i.e., on postoperative days (POD) 3 and 10 and on the day of discharge. After 3 months postsurgery, a follow-up examination is scheduled on an outpatient basis. During each visit at the delineated endpoints, the patient characteristics will be collected and recorded according to the CRF.

**Table 1:** Visit schedule

	Study period					
	Screening day of admission	Day of operation	POD 3	POD 10	Day of discharge	3 months after operation
Inclusion criteria	X					
Exclusion criteria	X	X				
Patient characteristics	X					
Randomization		X				
Used surgical technique		X				
Laboratory tests Morbidity			X	X	X	X

The study will collect baseline demographic data and information regarding the disease course (e.g., neoadjuvant therapy) and comorbidities from the included patients. During the postoperative study visits, routine blood tests (including hemoglobin concentration, leukocyte count, serum C-reactive protein, bilirubin, and liver and pancreatic enzymes) will be screened. Further, the amylase concentration in abdominal drains on the respective postoperative day (POD 3 and 10 or on the day of an intervention, e.g., CT-guided drainage) is recorded for identification and grading of a POPF. If no abdominal drains were inserted intraoperatively, the grading of a POPF is confined to the clinically relevant grades B and C (secondary endpoint) depending on the respective intervention or clinical status of the patient.

According to protocol, a CT scan of the abdomen or a CT angiography is not routinely performed during the study period. These diagnostic exams are indicated by the responsible physicians in each of the participating centers based on a medical rationale (e.g., suspected intra-abdominal fluid collection or hemorrhage, elevated liver enzymes or white blood cells/serum C-reactive protein). This management is considered standard in a certified pancreatic center. Further, symptomatic cardiorespiratory complications (e.g., pneumonia or myocardial infarction) are recorded based on routine diagnostic tests.

### Documentation and data management

All protocol-required information collected during this trial will be entered in the CRF. The completed CRFs will be reviewed, signed, and analyzed by the investigator or by a designated sub-investigator. During the trial, patients will be identified solely by means of their year of birth and individual identification code (screening number, randomization number; pseudonymized data). Trial findings will be stored in accordance with the local data protection law and GCP guidelines and will be handled in the strictest confidence. For the protection of these data, organizational procedures will be implemented to prevent the distribution of data to unauthorized people.

### Data monitoring and quality assurance

Data monitoring covers the inspection of CRFs along with original reports of the participating centers including the written operative notes. This also serves as a quality assurance for the study intervention. There is no mandatory photo documentation of the critical surgical study intervention (coverage of the GDA stump with the falciform ligament wrap) in all cases, because a standard image of the anatomic GDA site cannot reliably guarantee the complete and effective coverage of the GDA. However, the critical surgical steps (see “Surgical technique”) for performing the falciform ligament wrap and master photographs of completed wraps are taught by the head of the clinical trial during the study initiation. At least one intraoperative photo of the pedicled falciform wrap is obtained from each of the participating centers to check whether the applied technique is in line with the protocol. In addition, the anatomic and surgical characteristics of the falciform ligament wrap are requested in the intraoperative CRF (e.g., length of the ligament pedicle [<10 or ≥10 cm], type of GDA stump closure [per protocol or other], and length of the artery stump [<8 or ≥8 mm]).

There is no data monitoring committee (DMC) because the risk of the surgical intervention is considered very low according to the available literature. In case of medical or ethical rationales that advocate the continuation of the study (e.g., SAEs), the study can be stopped by the head of the clinical trial. Additional reasons are inadequate patient recruitment and additional external evidence recommending termination of the trial.

### Assessment of safety

Adverse (AEs) and serious adverse events (SAEs) will be documented within this trial. A SAE is defined as any adverse event that results in death, is life-threatening, requires or prolongs the hospitalization, or results in persistent or significant disability or incapacity.

SAEs that occur during the period between signature of the informed consent and 3 months after the operation are documented in the CRF. All SAEs must be documented on a “serious adverse event form.” The SAE form contains the following information: name of the attending physician, description of the SAE (event, beginning and duration, severity, outcome, causality to the trial intervention, therapy/interventions taken), consequence for the trial, and dated signature of the attending physician. SAEs have to be reported by the attending physician to the sponsor within 24 h after their occurrence.

### Ethical aspects

The trial is to be conducted in line with the Declaration of Helsinki. The study protocol was approved by the local ethical committee at the TU Dresden (decision number EK225062016). All local ethical committees of further centers have to approve the study before initiation. Before enrollment, the screened patients will be informed in detail about the aims and sequence of the study and furthermore about any possible risks and complications.

### Statistical considerations and sample size calculation

A comprehensive systematic literature review was performed and published by the authors in advance [12]. As a result, no prospective studies were identified which investigated a prophylactic round or falciform ligament wrap on the GDA stump for prevention of PPH. One retrospective study with a historical control group showed a significant reduction of postpancreatectomy bleeding by covering with an omental flap (*p* = 0.021; OR = 0.151; 95% CI, 0.030–0.751 [16]). The best available data on the falciform ligament wrap comes from China and was published in 2014. Xu et al. recently published a retrospective controlled review involving 140 patients per group, using the falciform ligament wrap as preventive intervention to reduce erosion hemorrhage [17]. There was one event in the intervention group, compared with nine events in the control group (incidence, 0.7 vs. 6.4%). The incidence of 6.4% seemed high, but an in-depth analysis of the own cohort supported that incidence [12]. The sample size estimation for the present trial was therefore based on the reduction of erosion hemorrhage rate from 6.4 to 0.7%. We used a two-tailed Fisher exact test for sample size calculation. To achieve an 80% power with a two-sided *p* value of less than 0.05, a group size of 174 patients is required. With a drop-out rate of 13%, the total sample size was calculated at 400 patients with 200 in each of the two groups.

Statistical analysis will be performed on an intention-to-treat and on a per-protocol-principle analysis. The Fisher exact test will be used to compare the different incidences of the primary and secondary endpoints. Statistical significance will be set at 0.05. The Student *t* test will be used to compare continuous variables (e.g., operation time), or alternatively the Mann-Whitney test. Statistical calculation will be done using the R statistics software package (R version 3.1.3, the R Foundation for Statistical Computing). The study protocol defines no interim analysis.

## Discussion

The clinical trial is the first randomized controlled study to investigate the effectiveness of a pedicled falciform ligament wrap for prevention of postpancreatectomy erosion hemorrhage. Creation of a falciform ligament wrap is a simple surgical technique with low associated morbidity. In the past, the falciform ligament has been used in hepatic injury [18] or for perforated duodenal ulcer [19]. More recently, it has been assessed for prevention of POPF after distal pancreatic resections [20]. The latter technique is at present analyzed by the randomized, controlled “DISCOVER” trial [21].

Compared with POPF, delayed erosion hemorrhage occurs less frequently but carries a high mortality [22]. The substantial mortality is closely related to the incidence of a POPF, formation of a pseudoaneurysm, delayed PPH, and soft pancreatic tissue [23]. Furthermore, pseudoaneurysm formation of the visceral vessels may be favored by the radicality of vascular dissection and lymphadenectomy, as well as by the length and closure of the GDA stump.

Before initiating the present trial, we performed a retrospective analysis of our own experience in using the pedicled falciform ligament wrap for patients undergoing PD. The study confirmed the high mortality of erosion hemorrhage (39%) and the feasibility of the technique. However, only 39 patients with the wrap were retrospectively identified, and the difference compared with cases without the wrap was not significant [12]. We then continued with a systematic literature review and found that the overall reported rate of erosion hemorrhage after PD and pedicled falciform ligament wrap is low (0.9%). In comparison, the median rate of erosion hemorrhage in studies which reported outcomes of PDs without the use of a wrap was 4.1 (interquartile range, 3.7–4.7) [8, 9, 23, 24].

The analysis of the own cohort also taught us that an exact differentiation of the site of erosion hemorrhage (GDA stump vs. HA) was not achieved in all cases, which is the reason why the definition of the endpoint of the randomized trial includes both sites in close vicinity. A further, recently published systematic review analyzed both the omental and the pedicled falciform ligament wrap to decrease the rate of POPF and PPH after PD [10]. The majority of the 12 included studies examined an omental flap for covering the visceral vessels or the pancreatic anastomosis. Only two (uncontrolled) studies were focused on the pedicled falciform ligament wrap for prevention of erosion hemorrhage [11, 25]. Consequently, the authors of the review concluded that randomized data are warranted to establish statistical evidence. Another retrospective questionnaire-based study from Japan reported that wrapping techniques with the omentum or falciform ligament did not reduce complications such as POPF and PPH [26]. However, the study has several limitations, such as the “survey” design of the study and the fact that the omental and falciform ligament wraps were not evaluated separately [25]. It still remains unclear whether there are different effects of the omental or falciform ligament wrap. For this reason, a prospective randomized trial should consider only one defined technique.

We decided to use the falciform ligament wrap, because more data are available for this technique and the creation of this wrap is easy to standardize. The pedicled falciform ligament flap is usually performed in 5–10 min. Longer operating times, which were observed in our own retrospective cohort of patients with wrap, were interpreted as a measure of the more complex cases with higher risk for POPF and PPH.

In conclusion, we outlined the potential importance of a prophylactic falciform ligament wrap to prevent erosion hemorrhage. We further summarized the paucity of the available data by performing a systematic literature review and meta-analysis. The initiation of the presented trial is the next logical step to generate increased evidence for evaluation of the effectiveness of the wrap in PD patients.

## Trial status

Recruitment is ongoing. The first patient was enrolled in July 2016.

## Additional file

Additional file 1: SPIRIT 2013 checklist: recommended items to address in a clinical trial protocol and related documents. (DOC 127 kb)

## Abbreviations

ASA: American Society of Anesthesiologists
CRF: Case report form
CT: computed tomography
GDA: Gastroduodenal artery
HA: Hepatic artery
PD: Pancreatoduodenectomy
PDS: Polydioxanone
POD: Postoperative day
POPF: Postoperative pancreatic fistula
PPH: Postpancreatectomy hemorrhage
