# Supplementary material for: Pancreatoduodenectomy with or without prophylactic falciform ligament wrap around the gastroduodenal artery stump for prevention of pancreatectomy hemorrhage
Source: Trials. 2018 Apr 12;19:222. doi: 10.1186/s13063-018-2580-0 (PMC5898061; doi:10.1186/s13063-018-2580-0)
Supplement: Supplementary file 1 — SPIRIT 2013 checklist: recommended items to address in a clinical trial protocol and related documents. (DOC 127 kb) [file 13063_2018_2580_MOESM1_ESM.doc]

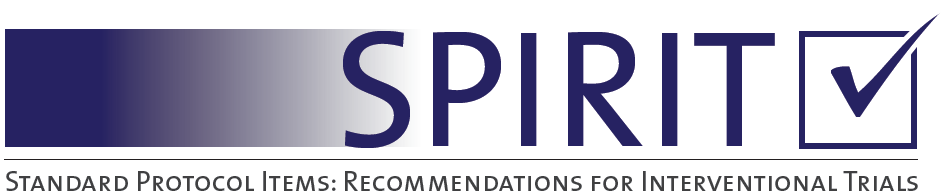


SPIRIT 2013 Checklist: Recommended items to address in a clinical trial protocol and related documents*

| Section/item | ItemNo | Description |
| --- | --- | --- |
| **Administrative information** | | |
| Title | 1 | Pancreatoduodenectomy with or without Prophylactic Falciform Ligament Wrap Around the Gastroduodenal Artery Stump for Prevention of Pancreatectomy Hemorrhage: study protocol for a randomized controlled trial |
| Trial registration | 2a | NCT02588066; http://www.clinicaltrials.org; October 27, 2015 |
|  | 2b | Not applicable |
| Protocol version | 3 | R1_12012018 |
| Funding | 4 | The trial is initiated by the Department of Visceral, Thoracic and Vascular Surgery, University Hospital Carl Gustav Carus, Technische Universität Dresden, Germany. Funding for this trial covers meetings and central organisational costs only; there is no third-party funding support for this trial. The contact information of the head of the clinical trial is: Fetscherstr. 74, 01307 Dresden, Germany (phone: +49 351 458 2742, fax: +49 351 458 7240, email: klinikportal-vtg@uniklinikum-dresden.de). The head of the trial is the senior author of this study protocol. All participating centers have to sign a collaboration contract, which regulates responsibilities, ownership and publication issues with the head of the clinical trial. |
| Roles and responsibilities | 5a | B.M. collected and analyzed own previous data and literature, and drafted the manuscript.  L.Z. and A.W. assisted with the data collection.  J.W., N.N.R. and M.D. worked on the study design and the final manuscript.  D.S. assisted with study design, organization of study visits and monitoring.  T.W. designed the study, collected and analyzed the data, and finalized the manuscript. The statistical considerations were supervised by N.N.R. and T.W.  X.G. has reviewed the manuscript. All authors read and approved the final manuscript. |
|  | 5b | The trial is initiated by the Department of Visceral, Thoracic and Vascular Surgery, University Hospital Carl Gustav Carus, Technische Universität Dresden, Germany. The contact information of the head of the clinical trial is: Fetscherstr. 74, 01307 Dresden, Germany (phone: +49 351 458 2742, fax: +49 351 458 7240, email: klinikportal-vtg@uniklinikum-dresden.de). |
|  | 5c | See above (5a) |
|  | 5d | The head of the trial is the senior author of this study protocol. All participating centers have to sign a collaboration contract, which regulates responsibilities, ownership and publication issues with the head of the clinical trial. |
| Introduction |  |  |
| Background and rationale | 6a | The purpose of this study is to evaluate whether wrapping of the pedicled falciform ligamentum flap around the gastroduodenal artery (GDA) stump/ hepatic artery can significantly decrease the incidence of erosion hemorrhage after pancreatoduodenectomy (PD).  Ramia JM, de la Plaza R, Adel F, Ramiro C, Arteaga V, Garcia-Parreno J: Wrapping in pancreatic surgery: a systematic review. ANZ journal of surgery 2014, 84(12):921-924.  Sakamoto Y, Shimada K, Esaki M, Kajiwara T, Sano T, Kosuge T: Wrapping the stump of the gastroduodenal artery using the falciform ligament during pancreaticoduodenectomy. Journal of the American College of Surgeons 2007, 204(2):334-336.  Mussle B, Wierick A, Distler M, Weitz J, Welsch T: Falciform ligament wrap for prevention of gastroduodenal artery bleed after pancreatoduodenectomy. The Journal of surgical research 2017, 207:215-222.  Xu C, Yang X, Luo X, Shen F, Wu M, Tan W, Jiang X: "Wrapping the gastroduodenal artery stump" during pancreatoduodenectomy reduced the stump hemorrhage incidence after operation. Chinese journal of cancer research = Chung-kuo yen cheng yen chiu 2014, 26(3):299-308.  Abe N, Sugiyama M, Yanagida O, Masaki T, Mori T, Atomi Y: Wrapping of skeletonized and divided vessels using the falciform ligament in distal pancreatectomy. American journal of surgery 2007, 194(1):94-97.  Tani M, Kawai M, Hirono S, Hatori T, Imaizumi T, Nakao A, Egawa S, Asano T, Nagakawa T, Yamaue H: Use of omentum or falciform ligament does not decrease complications after pancreaticoduodenectomy: nationwide survey of the Japanese Society of Pancreatic Surgery. Surgery 2012, 151(2):183-191. |
|  | 6b | Not apllicable |
| Objectives | 7 | The pedicled falciform ligament wrap can significantly decrease the incidence of postpancreatectomy erosion hemorrhage. |
| Trial design | 8 | The study is designed as a randomized controlled, national (Germany) multi-center trial with an interventional group (A: PD operation with the creation of a pedicled falciform ligament wrap around the GDA stump), and a control group (B: PD operation without the ligament wrap). |
| Methods: Participants, interventions, and outcomes | | |
| Study setting | 9 | Participating centers are at least 4 German university centers and two academic centers, which are officially certified for pancreatic surgery (pancreatic cancer center). Immediate access to computed tomography (CT) and a 24-hour availability of interventional radiology must be guaranteed. A list of participating centers can be obtained from the senior author by request. |
| Eligibility criteria | 10 | The targeted study population includes all patients scheduled for elective open PD (Whipple or pylorus-preserving) with reconstruction using a pancreatojejunostomy (anastomosis of the pancreas to the jejunum) in cases of tumors or cystic lesions of the pancreatic head and distal bile duct, the duodenum, or in cases of chronic pancreatitis. Further inclusion criteria are: male and female patients, age equal to or older than 18 years, American Society of Anesthesiologists (ASA) score I-III, and a completed written informed consent form. Exclusion criteria are: potential conditions/circumstances after previous abdominal surgery with resection of falciform ligament (e.g. status post liver resection), no creation of pancreatojejunostomy (e.g. pancreatogastrostomy, total pancreatectomy, or non-resectability), simultaneous arterial resection or reconstruction (e.g. hepatic or splenic, or superior mesenteric artery). |
| Interventions | 11a | The technique for creation of the pedicled falciform ligament wrap as it should be performed in this trial, is as follows:  After completion of the pancreatic, bile duct or gastric/duodenal anastomosis, the prepared pedicled falciform ligament is carefully tunneled below the common HA and wrapped around the GDA stump in a tension-free fashion using only one turn. Fixation is then performed with 2–3 stitches using polydioxanone (PDS) 5-0. The last step is to ensure a proper pulsation of the HA after completion of the wrap. |
|  | 11b | In cases when exclusion criteria (e.g. no performance of a pancreatojejunostomy or total pancreatectomy) are met after randomization, the respective patients were withdrawn from the trial. |
|  | 11c | Not applicable |
|  | 11d | The study will collect baseline demographic data and information regarding the disease course (e.g., neoadjuvant therapy) and comorbidities from the included patients. During the postoperative study visits, routine blood tests (including hemoglobin concentration, leukocyte count, serum C-reactive protein, bilirubin, liver and pancreatic enzymes) will be screened. Further, the amylase concentration in abdominal drains on the respective postoperative day (postoperative days 3 and 10, or at the day of an intervention, e.g., CT-guided drainage) is recorded for identification and grading of a POPF. If no abdominal drains were inserted intraoperatively, the grading of a POPF is confined to the clinically relevant grades B and C (secondary endpoint) depending on the respective intervention or clinical status of the patient.  According to protocol, a CT scan of the abdomen or a CT angiography is not routinely performed during the study period. These diagnostic exams are indicated by the responsible physicians in each of the participating centers based on a medical rationale (e.g., suspected intraabdominal fluid collection or hemorrhage, elevated liver enzymes or white blood cells/ serum C-reactive protein). This management is considered standard in a certified pancreatic center. Further, symptomatic cardiorespiratory complications (e.g., pneumonia or myocardial infarction) are recorded based on routine diagnostic tests. |
| Outcomes | 12 | The aim of the study is to evaluate whether a pedicled falciform ligament wrap can decrease erosion hemorrhage of the stump of the GDA in the presence of a POPF.  Therefore, the primary endpoint of the study is rate of postoperative erosion hemorrhage from the GDA stump or the HA within 3 months from the index operation.  Secondary endpoints are:  Incidence of late PPH (according to the International Study Group of Pancreatic Surgery [ISGPS ] Definition [13]) in the entire study population and divided into subgroups with soft and hard pancreatic texture (also depending on the underlying histopathology [e.g. cancer, cystic lesion or chronic pancreatitis])  Incidence of clinically relevant POPF (according to POPF grades B and C, ISGPS definition, [3, 14])  Incidence of symptomatic hepatic malperfusion and narrowing/stenosis of the HA (diagnosed by computed tomography or angiography)  Postoperative rate of therapeutic interventions (computed tomography-guided drainage or angiographic studies)  Reoperation rate  Postoperative morbidity and mortality (during hospital stay and at 3 months postoperative). |
| Participant timeline | 13 | The trial includes a total of 5 study visits during the operation or the postoperative period. The period ranges from the day of the operation until 3 months after the operation (Table 1). All outcome parameters will be recorded by a surgical resident or fellow before and after the operation, i.e. on postoperative days (POD) 3, 10, and on the day of discharge. After three months post surgery, a follow-up examination is scheduled on an outpatient basis. During each visit at the delineated endpoints, the patient characteristics will be collected and recorded according to the case report form (CRF). |
| Sample size | 14 | A comprehensive systematic literature review was performed and published by the authors in advance. As a result, no prospective studies were identified which investigated a prophylactic round or falciform ligament wrap on the GDA stump for prevention of PPH. One retrospective study with a historical control group showed a significant reduction of post pancreatectomy bleeding by covering with an omental flap (p=0.021; OR= 0.151; 95 % CI, 0.030-0.751). The best available data on the falciform ligament wrap come from China and was published in 2014. Xu et al. recently published a retrospective controlled review involving 140 patients per group, using the falciform ligament wrap as preventive intervention to reduce erosion hemorrhage. There was one event in the intervention group, compared with 9 events in the control group (incidence: 0.7 % vs. 6.4 %). The incidence of 6.4 % seemed high, but an in-depth analysis of the own cohort supported that incidence. The sample size estimation for the present trial was therefore based on the reduction of erosion hemorrhage rate from 6.4 % to 0.7 %. We used a 2-tailed Fisher Exact test for sample size calculation. To achieve an 80 % power with a 2-sided P value of less than 0.05, a group size of 174 patients is required. With a drop-out rate of 13 %, the total sample size was calculated at 400 patients with 200 in each of the two groups. |
| Recruitment | 15 | Multicenter trial. Patients will be screened for eligibility considering the inclusion criteria on the day of admission (usually the day before the surgery). |
| **Methods: Assignment of interventions (for controlled trials)** | | |
| Allocation: |  |  |
| Sequence generation | 16a | Randomization will be performed intraoperatively after proving the exclusion criteria. The randomization is designed as block randomization (via envelopes), with fixed block sizes in a 1:1 allocation ratio. |
| Allocation concealment mechanism | 16b | The envelopes were prepared by an authorized trial coordinator at the center in Dresden and distributed to the participating sites as needed. It is mandatory to check all eligibility criteria before opening of a randomization envelope by authorized trial personnel only. The investigator has to consecutively assign the envelopes to the patients. He is requested to document the assignment carefully in the patient identification log.  The details of randomization will be kept safe and confidential. Subjects withdrawn from the trial retain their identification codes (e.g. randomization number). New subjects receive a new identification code. |
| Implementation | 16c | By an authorized trial coordinator, by the investigator or by a designated sub-investigator |
| Blinding (masking) | 17a | Not applicable |
|  | 17b | Not applicable |
| **Methods: Data collection, management, and analysis** | | |
| Data collection methods | 18a | The trial includes a total of 5 study visits during the operation or the postoperative period. The period ranges from the day of the operation until 3 months after the operation (Table 1). All outcome parameters will be recorded by a surgical resident or fellow before and after the operation, i.e. on postoperative days (POD) 3, 10, and on the day of discharge. After three months post surgery, a follow-up examination is scheduled on an outpatient basis. |
|  | 18b | During each visit at the delineated endpoints, the patient characteristics will be collected and recorded according to the case report form (CRF). |
| Data management | 19 | All protocol-required information collected during this trial will be entered in the CRF. The completed CRFs will be reviewed, signed and analyzed by the investigator or by a designated sub-investigator. During the trial, patients will be identified solely by means of their year of birth and individual identification code (screening number, randomization number; pseudonymized data). Trial findings will be stored in accordance with the local data protection law and GCP guidelines, and will be handled in the strictest confidence. For the protection of these data, organizational procedures will be implemented to prevent the distribution of data to unauthorized people. |
| Statistical methods | 20a | Statistical analysis will be performed on an intention-to-treat and on the per-protocol-principle analysis. The Fisher Exact test will be used to compare the different incidences of the primary and secondary endpoints. Statistical significance will be set at 0.05. The Student-t test will be used to compare continuous variables (e.g. operation time), or, alternatively the Mann-Whitney test. Statistical calculation will be done using the R statistics software package (R version 3.1.3, the R Foundation for Statistical Computing).The study protocol defines no interim analysis. |
|  | 20b | See above |
|  | 20c | Not applicable |
| **Methods: Monitoring** | | |
| Data monitoring | 21a | There is no data monitoring committee (DMC) because the risk of the surgical intervention is considered very low according to the available literature. In case of medical or ethical rationales that advocate the continuation of the study (e.g. SAEs) the study can be stopped by the head of the clinical trial. Additional reasons are inadequate patient recruitment and additional external evidence recommending termination of the trial. |
|  | 21b | The study protocol defines no interim analysis. |
| Harms | 22 | Adverse (AEs) and serious adverse events (SAE) will be documented within this trial.  A SAE is defined as any adverse event that results in death, is life-threatening, requires or prolongs the hospitalization or results in persistent or significant disability or incapacity.  Serious adverse events that occur during the period between signature of the informed consent and 3 months after the operation are documented in the CRF. All SAE must be documented on a “serious adverse event form”. |
| Auditing | 23 | Not applicable |
| Ethics and dissemination | | |
| Research ethics approval | 24 | The trial is to be conducted in line with the Declaration of Helsinki. The study protocol was approved by the local Ethical Committee at the TU Dresden (decision number EK225062016). All local Ethical Committees of further centers have to approve the study before initiation. |
| Protocol amendments | 25 | Not applicable |
| Consent or assent | 26a | Before enrollment, the screened patients will be informed in detail about the aims and sequence of the study by the investigator or by a designated sub-investigator, and furthermore about any possible risks and complications. |
|  | 26b | Not applicable |
| Confidentiality | 27 | Personal information about enrolled participants will be collected in the CRF form, and maintained in an database |
| Declaration of interests | 28 | The authors declare that they have no competing interests. |
| Access to data | 29 | The head of the clinical trial and the investigators |
| Ancillary and post-trial care | 30 | Not applicable |
| Dissemination policy | 31a | We publish the results of the described clinical trial. |
|  | 31b | As listed in the protocoll |
|  | 31c | Not applicable |
| Appendices |  |  |
| Informed consent materials | 32 | Model consent form and other related documentation given to participants and authorised surrogates |
| Biological specimens | 33 | Not applicable |

*It is strongly recommended that this checklist be read in conjunction with the SPIRIT 2013 Explanation & Elaboration for important clarification on the items. Amendments to the protocol should be tracked and dated. The SPIRIT checklist is copyrighted by the SPIRIT Group under the Creative Commons “[Attribution-NonCommercial-NoDerivs 3.0 Unported](http://www.creativecommons.org/licenses/by-nc-nd/3.0/)” license.
